# Management of Dupuytren disease of the little finger

**DOI:** 10.1186/s13018-025-06176-2

**Published:** 2025-08-22

**Authors:** Melinda Moscovici, Valerio Pace, Fabrizio Marzano, Francesco Bronzini, Giacomo Placella, Dario Perugia, Nicola Maffulli, Filippo Migliorini, Riccardo Maria Lanzetti

**Affiliations:** 1 Université Grenoble Alpes (UGA), Grenoble, Saint-Martin-d’Hères 38400 France; 2 Department of Trauma and Orthopaedics, “MVT-Pantalla” Hospital, ULS Umbria 1, Umbria, 06059 Italy; 3 Department of Trauma and Orthopaedics, University of Perugia, Perugia, 06100 Italy; 4 San Raffaele Hospital Università (IRCCS), Milan, 20132 Italy; 5 Faculty of Medicine and Psychology, University La Sapienza, Rome, 00185 Italy; 6 School of Pharmacy and Bioengineering, Faculty of Medicine, Keele University, Stoke On Trent, ST4 7QB UK; 7 Centre for Sports and Exercise Medicine, Barts and the London School of Medicine and Dentistry, Mile End Hospital, Queen Mary University of London, London, E1 4DG UK; 8 Department of Trauma and Reconstructive Surgery, University Hospital of Halle, Martin-Luther University Halle-Wittenberg, Ernst-Grube-Street 40, Halle (Saale), 06097 Germany; 9 Department of Orthopaedic and Trauma Surgery, Academic Hospital of Bolzano (SABES-ASDAA), Via Lorenz Böhler 5, Bolzano, 39100 Italy; 10 Department of Life Sciences, Health, and Health Professions, Link Campus University, Via del Casale Di San Pio V, Rome, 00165 Italy; 11 Orthopaedics and Traumatology Unit, Department of Emergency and Acceptance, Hospital San Camillo-Forlanini, Rome, 05152 Italy

**Keywords:** Dupuytren's contracture, Dupuytren’s disease, Little finger, Little finger contracture, Hand surgery

## Abstract

**Background:**

The surgical management of Dupuytren disease (DD) is associated with a high rate of complications. Recurrences are relatively common and may result in permanent disability, particularly when the little finger (LF) is involved. This study aims to provide both objective and subjective information, along with professionals’ experiences.

**Methods:**

A questionnaire survey, comprising both open and closed questions, was distributed to hand surgeons, physiotherapists, and occupational therapists engaged in the management of DD across five continents. The involvement and role of the LF in DD were extensively highlighted and emphasised. Only consistent answers were included. A total of 588 questionnaires were completed.

**Results:**

50% (*n* = 294) of the answers were from hand surgeons, 24% (*n* = 141) from physiotherapists and 26% (*n* = 153) from occupational therapists. 76.5% (*n* = 153) of the healthcare professionals (HCP) agreed that: “The LF does not necessarily benefit from good results. Rehabilitation, just like surgery, can be delicate and difficult.”. Different agreements were found between surgeons and occupational therapists (*p* = 0.007) and among surgeons, depending on their surgical experience (*p* = 0.008). No significant differences were seen between surgeons and physiotherapists.

**Conclusions:**

The LF in Dupuytren’s disease requires special attention during surgery and rehabilitation. All healthcare professionals (HCPs) should invest in patient education to ensure early referral and optimal adherence to treatments. Further high-quality research is warranted to achieve a definitive consensus on optimal treatment and rehabilitation.

**Supplementary Information:**

The online version contains supplementary material available at 10.1186/s13018-025-06176-2.

## Background

Dupuytren’s disease (DD) affects the palmar fascia and can lead to pronounced flexion contracture and disability. Recurrence and postoperative complications are common and often challenging to manage [1]. Dupuytren’s contracture frequently affects the ulnar digits, resulting in significant impairment of hand grip and function. The cause of Dupuytren contracture remains unknown, although a familial component has been reported in several studies. It is also noted that DD is more prevalent in men than in women [2]. DD affecting the little finger (LF) presents management challenges due to the presence of the abductor digiti minimi (ADM), the risk of iatrogenic injury, and a recurrence rate ranging from 16.7% to 39.4%, depending on surgical technique and follow-up duration [3, 4]. Several studies indicate that the most challenging DD cases occur when it affects the little finger [5]. The contracture of the proximal interphalangeal joint (PIPJ) can be severe and complex to correct and is generally more problematic for the LF than for the other fingers [6, 7]. The PIPJ is unforgiving and may progress to non-functional arthrodesis after prolonged involvement, necessitating amputation in some patients [8]. Alternative surgical treatments for DD of the LF do not always guarantee favourable functional or aesthetic outcomes [9, 10]. There remains a lack of scientific evidence regarding the optimal approach and treatment algorithm for DD affecting the LF, indicating substantial room for improvement. Further research with a high level of evidence is certainly needed in this field. The scarcity of knowledge and evidence presents a real challenge for surgeons dealing with such heterogeneous and complex conditions. Furthermore, the outcomes of surgical treatment options are often reported as less than satisfactory, with a high rate of complications and recurrence [7–10].

A multidisciplinary questionnaire was developed and administered to professionals handling cases of developmental disabilities (DD) affecting the lower limb (LF). The primary aim of this study was to provide both objective and subjective information, along with professionals’ experiences in managing DD involving the LF, to shed light on current knowledge regarding management options, rehabilitation, and outcomes, as well as to offer insights for future research. The secondary aim of this study was to identify gaps in the literature and gather data on the challenges faced by professionals in managing the LF in cases of DD.

## Methods

A multidisciplinary questionnaire was developed by a diverse team with expertise in managing DD. Surgeons, physiotherapists, and occupational therapists working in hand surgery worldwide were asked both open and closed questions. The full text of the survey can be found in Appendix 1. It gathered pertinent information about the surgeon or therapist being questioned, including their country of origin, occupation type (surgeon, physiotherapist, etc.), the average number of patients with DD treated each year, the average number of patients with DD affecting the LF treated annually, the number of isolated little finger contractures, agreement with the statement “5th finger does not necessarily benefit from good results. Rehabilitation, just like surgery, can be delicate and difficult" along with an explanation for their answer, and interest in knowing the questionnaire results. The keyword coding tables and word clouds used are detailed in Appendix 2. The survey was created and distributed via Google Forms over a set timeframe of six months. It was disseminated through professional channels to healthcare professionals. To maximise response rates, the survey was available in both English and French, allowing respondents to answer in their preferred language. Responses were subsequently translated using the free DeePL Translator (https://www.deepl.com/translator). The survey was shared on social media platforms (Facebook, LinkedIn, Twitter) and through personal networking. Each question required a mandatory response to progress to the next one and complete the questionnaire. Incongruous answers that attempted to bypass the requirement to answer each question were excluded.

The survey was based on the following key statement: “The little finger does not necessarily benefit from good results. Rehabilitation, just like surgery, can be delicate and difficult. Do you agree with this point of view?”. To quantify the practitioners’ experience, the column "number of patients with Dupuytren Disease treated in 1 year" was used, with the following mapping: 0–15, low volume; 15–30, intermediate volume; and > 30, high volume. Participants were asked to agree or disagree with the statement and provide an explanation for their response, specifically about their professional field. The distribution of answers was analysed by country and professional experience. Participants were asked to enter their country of work and clarify their professional experience, specifying the number of DD patients they treated on average over the past year. Moreover, the frequency of isolated LF involvement was reported. Current knowledge and evidence on the treatment and rehabilitation of DD involving the LF were also searched on PubMed and Cochrane databases, to be then analysed, integrated, and discussed concerning our results. We therefore highlighted the level of evidence and the lack of evidence of the main related aspects, underlining the weaknesses and debates still to be resolved.

Data analysis was performed using Microsoft Excel and Python. T-test and Chi-Square calculations were performed. Statistical significance was set at *p* < 0.05.

## Results

**Fig. 1:**
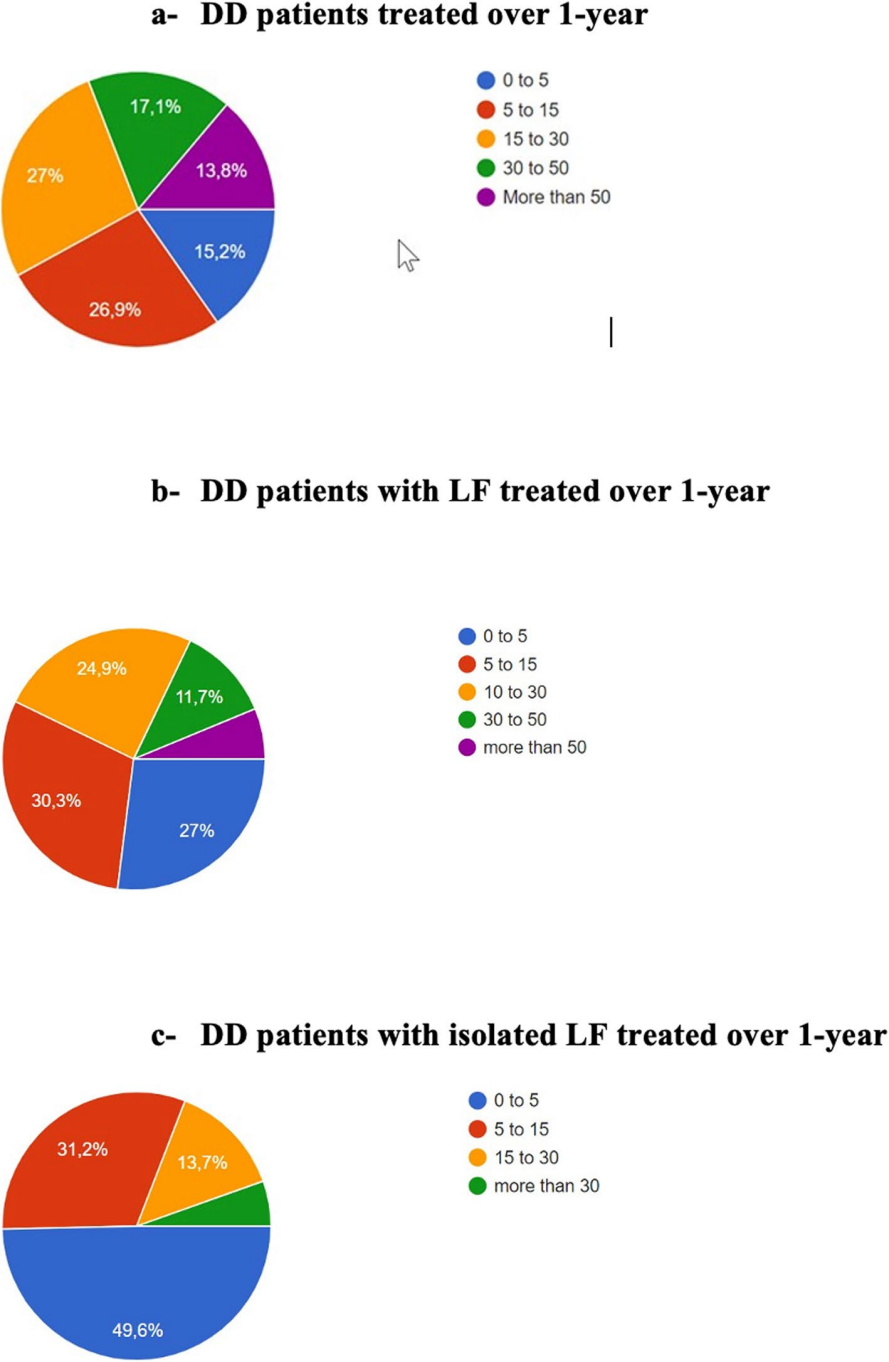
The number of DD patients treated over the course of one year with LF (**a**) or isolated LF (**b**)

**Fig. 2:**
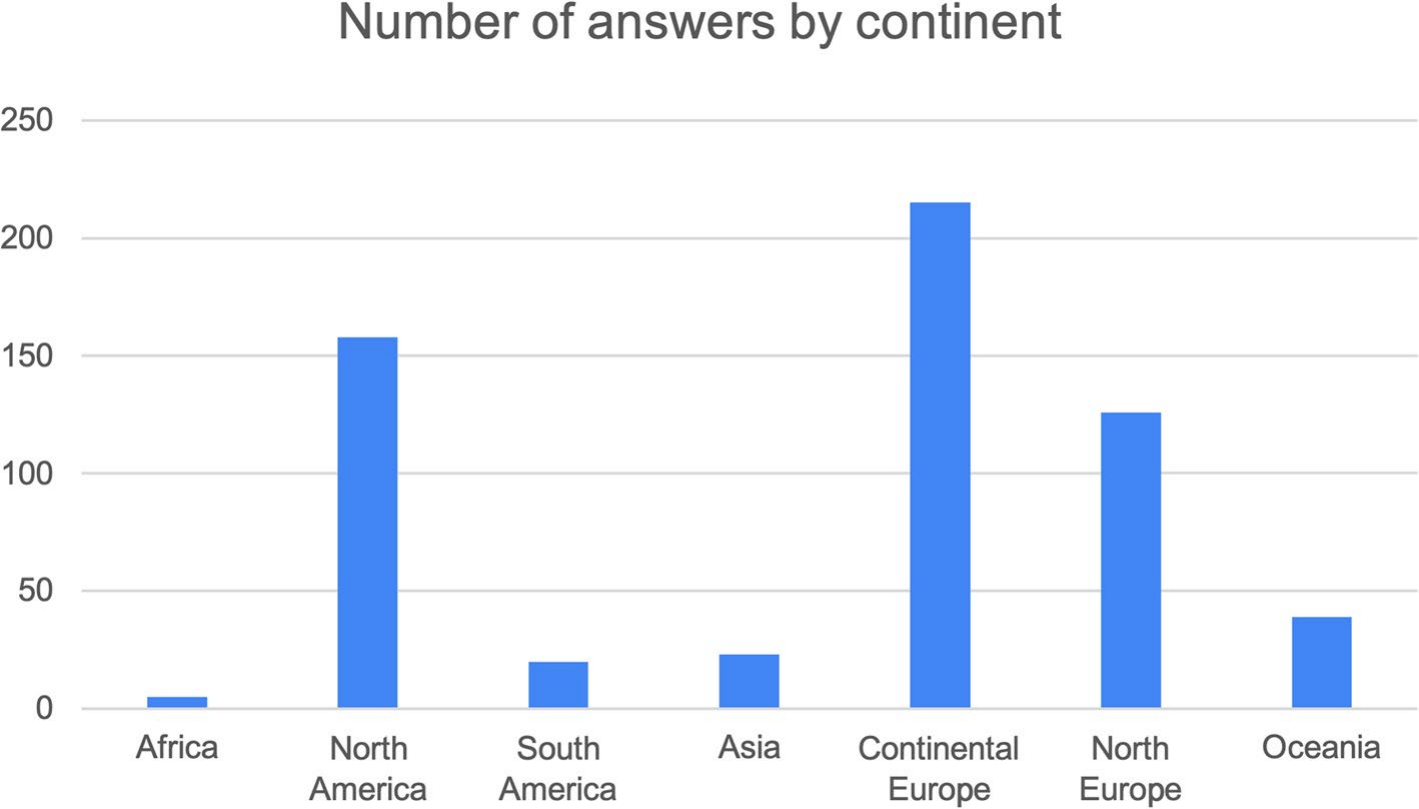
The Number of answeres by various continent

**Fig. 3:**
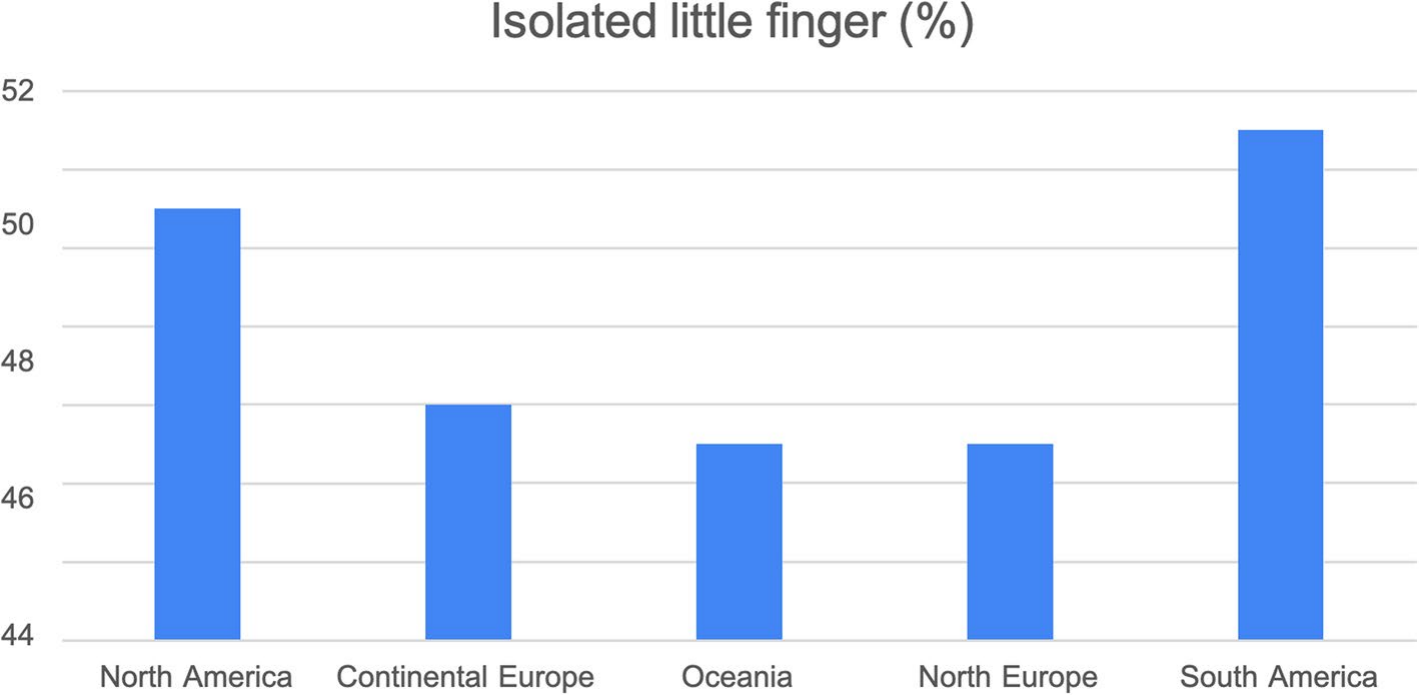
The percentage of isolated DD of the LF by continent

**Fig. 4:**
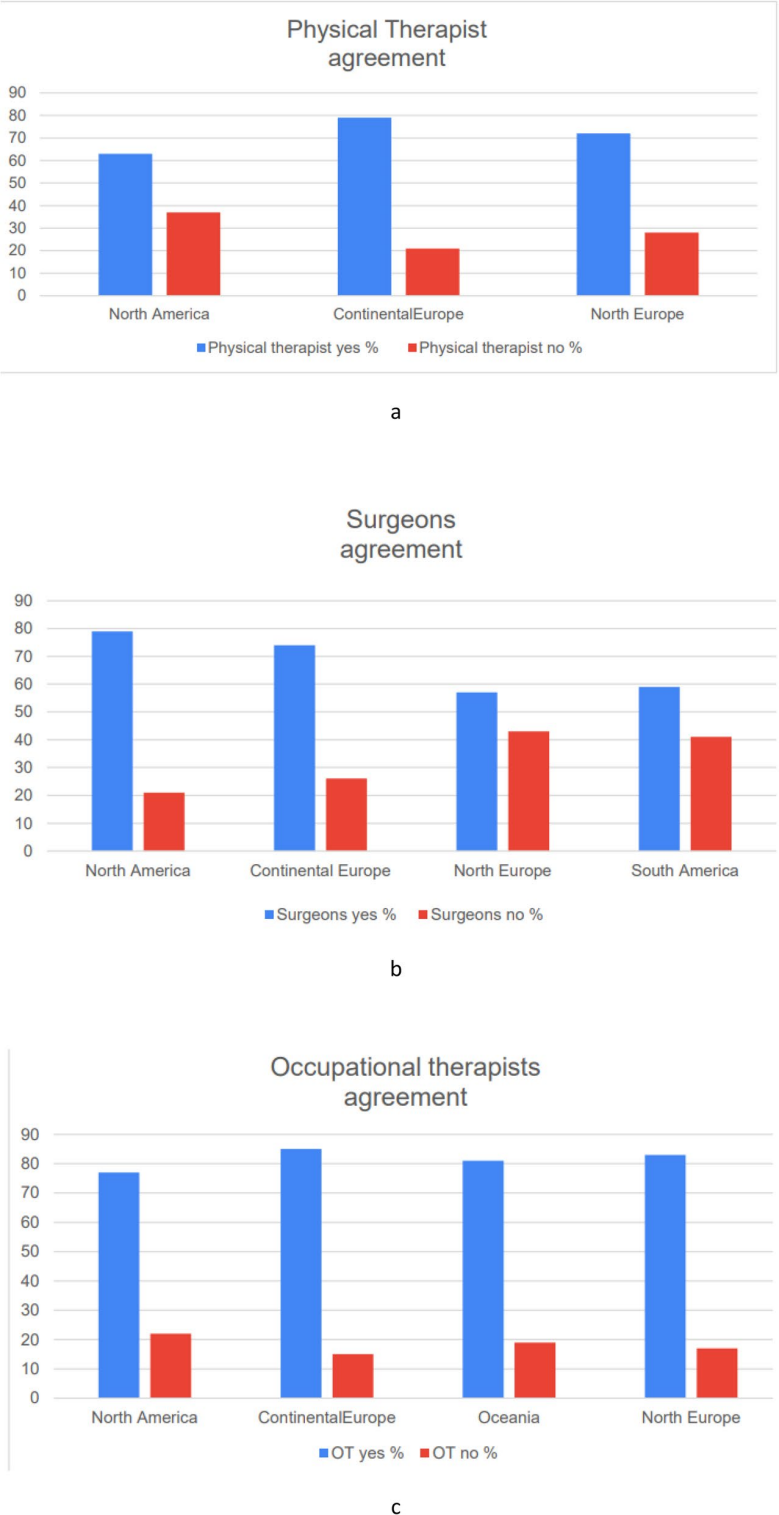
The percentage of agreement (yes) and disagreement (no) among physical therapists, surgeons, and occupational therapists

We distributed the survey to 1,000 HCPs and received a total of 588 responses: 50% (294) from hand surgeons, 24% (141) from physiotherapists, and 26% (153) from occupational therapists. The number of DD patients treated over the course of one year with LF or isolated LF is summarised in Fig. 1 a-c. The responses obtained from various countries were grouped by continent: Africa, North America, South America, Asia, Continental Europe, Northern Europe, and Oceania (Fig. 2). We observed the percentage of isolated DD of the LF by continent to provide a general idea of the prevalence of isolated LF (Fig. 3). Regarding the key statement (Question 6), 75.7% (445) of professionals agreed. 445 of the participants in the survey agreed with the key main statement: “The LF does not necessarily benefit from good results. Rehabilitation, just like surgery, can be delicate and difficult.” Most responses came from North America, Oceania, and Europe (Continental and Northern Europe) (Fig. 2). To observe the distribution of agreement and disagreement with the statement in Question 6 by profession and continent, we only included continents with a minimum of 15 responses. Figure 4 a-c shows the percentage of agreement (yes) and disagreement (no) among physical therapists, surgeons, and occupational therapists.

The T-test revealed significant differences among professional categories concerning agreement with the key statement (*p* = 0.019). A notable difference existed between hand surgeons and occupational therapists who concurred with the statement (*p* = 0.007). Additionally, a significant difference was observed among surgeons based on their level of experience (*p* = 0.008). There was no significant difference between surgeons and physical therapists (*p* = 0.28) or between physical therapists and occupational therapists (*p* = 0.184). No significant difference was noted among physical therapists (*p* = 0.416) and occupational therapists (*p* = 0.754) based on their experience level. The most frequently mentioned words by participants who disagreed with the statement are presented in descending order for each professional category. Similarly, the most commonly mentioned words used by participants who agreed with the statement are also presented in descending order for each professional category (Supplementary Material: Table 2 a-b).

## Discussion

The most important finding of the present investigation is that the DD of the LF needs special attention both during surgery and rehabilitation. All HCPs should invest in patient education to ensure early referral and optimal treatment adherence. Further high-quality research is warranted to achieve a definitive consensus on optimal treatment and rehabilitation.

The prevalence of Dupuytren's Disease (DD) ranges from 3 to 42% in the adult population [3–5]. The prevalence of isolated LF’s DD is around 45%, which is higher than expected given the published evidence [11–13]. The LF is the second most commonly affected finger in DD after the ring finger, and managing DD in this location can be challenging. The fibrous contracture of the palmar fascia often includes the tendon of the abductor digiti minimi (ADM), and this process leads to both a flexion contracture at the MCP joint and an abduction contracture [6, 9, 14]. The functional outcomes of managing DD in the LF depend on the degree of the PIP joint contracture. All healthcare professionals (HCPs) concur that PIPJ contracture is the principal factor contributing to the difficulty in managing LF DD, whether through surgical or rehabilitative means. The contracture of the PIPJ tends to persist even after surgical release and rehabilitation. The recurrence rate of DD in the LF ranges from 0 to 100%, indicating a high recurrence rate [15–17]. Physiotherapists (PTS) and occupational therapists (OTS) tend to use the term “chronic” more frequently than surgeons to describe a residual and persistent condition. In contrast, surgeons emphasise the concept of recurrence. Recurrence refers to the formation of a new cord following excision, while “chronic” denotes the duration of the condition according to surgeons. In this context (as all professionals mentioned), the LF continues to exhibit a range of motion (ROM) deficit in both flexion and extension. More than 20 degrees of contracture is considered recurrence in any treated joint one year post-treatment, as compared to six weeks post-treatment. Recurrence appears to be less common if good correction is attained during surgery [15]. However, no surgical technique demonstrates more favourable recurrence rates [18–24].

Another significant factor highlighted by the three professions is skin complications [25, 26]. During surgery, skin deficiency on the LT often necessitates the use of skin grafts to reduce the risk of suturing skin under tension and to avoid neurovascular complications. In rehabilitation, slower skin healing, scar adhesion, and denser, more prominent scar tissue over a small area frequently restrict movement. Furthermore, surgeons refer to both rehabilitation and surgery. Rehabilitation is deemed essential for a successful outcome, yet it is simultaneously blamed for unsatisfactory results. A lack of hand therapists and rehabilitation protocols has been identified as potential causes. Concerning surgery, the issues raised relate to the timing of the operation, the selection of the appropriate technique, the challenge of releasing and excising all the pathological tissue in a small area, and the difficulty in maintaining surgical correction postoperatively. A crucial point emphasised by physical therapists is finger exclusion. In the LF, exclusion seems linked to the marginal position of the finger, making it easier to avoid using in daily activities. Finger exclusion is detrimental both pre- and post-surgery. Before surgery, it distorts patients’ perception of the problem, resulting in delayed consultations and poor compliance after surgery. This undermines postoperative rehabilitation, as patients are less cooperative with treatment, and a finger not integrated into the motor schema will create greater difficulty in achieving correct motion. Occupational therapists highlight extension deficit and secondary contracture. The primary reasons could be: (1) the tendency for the MCPJ of the LF to hyperextend; (2) chronic stretching of the extensor tendon, leading to active extensor lag even when fully released; (3) contracture of the flexor tendons and volar periarticular structures, resulting in increased force required to achieve extension. Professionals who disagreed with the statement argued that the management of DD of the LF yields good results and patient satisfaction when there is strong patient compliance and early intervention with a lesser degree of PIP joint involvement. Good surgical skills have also been cited as prerequisites for positive outcomes. Unlike other professions, surgeons demonstrate marked differences in their responses, depending on their experience level. Less experienced surgeons tend to be more reluctant to assert that surgery on the LF in DD is more challenging. Multidisciplinary treatment guidelines, developed through a European Delphi consensus strategy, agreed that a surgeon’s experience is a crucial factor in selecting the surgical technique [27].

There is no evidence regarding the relative superiority of needle and open fasciotomy, as well as limited fasciectomy and dermofasciectomy [28–30]. However, experts agree that considerable experience is required, regardless of the technique. The influence of a surgeon’s experience on the choice of surgical technique may be even more apparent in more complex surgeries. Ullah et al. concluded that since skin grafting is more likely to be performed by a senior surgeon, the lower rate of recurrence could be associated with a more expert and complete excision of the contracted fascia [20]. Incomplete correction of a PIPJ deformity increased the likelihood of worse postoperative contracture [10]. However, the greater the surgical correction, the greater the chance of losing some of that correction at follow-up [31, 32]. A complete release may be harmful and unnecessary, especially when a given technique is not well mastered.

It is crucial to reintegrate the LF into the motor scheme through activities of daily living to promote its functional use. Motor imagery may be helpful in patients with painful and stiff fingers. Patients should incorporate the use of LF into their daily life to remain compliant with their treatment. Regarding splinting, difficulty in managing the short lever on the LF was mentioned, as well as the lack of established splinting protocols. There is still no consensus, despite a tendency to use static over dynamic splints having emerged. A palmar splint could be preferable to a dorsal splint. Isolated full extension of the long extensors of the fingers results in hyperextension of the MP joints but incomplete extension of the PIP and DIP joints [33, 34]. A Yoke splint that prevents MP hyperextension could allow the long extensors and the intrinsic muscles to fully extend the IP joints.

There is a delicate balance between the surgical and functional outcomes in the LF in DD. Functional improvement should be the primary purpose of corrective DD surgery. The anatomy and cortical representation are different in each finger. Expected outcomes after treatment vary from individual to individual. Any research should not only measure a range of motion but also the effect of that intervention on hand function [35, 36]. This implies using a common functional outcomes measure that best fits the specific needs of DD patients. To our knowledge, such a PROM (patient-reported outcome measure) has not yet been developed.

This study aimed to collect data on the difficulties faced by professionals in managing the LF in DD. The present study does carry limitations. For example, distributing the questionnaire through social media may have biased the participant population, favouring those who are more accustomed to using such media. The questionnaire did not collect data on age, seniority in practice, place of professional activity (hospital specialised in hand surgery or rehabilitation clinic), and educational background (whether participants have specialised hand surgery training). The limited number of answers and their geographic distribution may have been influenced by the ease of access to European professionals compared to those on other continents. Nevertheless, the present investigation may form the basis for further research to translate these preliminary findings into actionable advice for surgeons and therapists, ultimately leading to an international consensus on the optimal treatment and rehabilitation for DD of the LF. Surgeons should be aware of the anatomical and functional peculiarities of DD and the involvement of the LF in DD. In addition, they should be mindful that surgery in DD, though common, can be technically demanding. Physical and occupational therapists play a key role in optimising surgical outcomes and the early detection and management of postoperative complications. The effectiveness of splinting should be investigated further. Additionally, rehabilitation should focus on the cortical reintegration of the LF. Education of general practitioners and patients is to be promoted to ensure early referral and better compliance with treatment and rehabilitation.

## Supplementary Information

Supplementary Material 1.

## Data Availability

The datasets generated during are available under reasonable request to (valeriopace@doctors.org.uk).
