# Supplementary material for: Management of Dupuytren disease of the little finger
Source: J Orthop Surg Res. 2025 Aug 22;20:789. doi: 10.1186/s13018-025-06176-2 (PMC12374359; doi:10.1186/s13018-025-06176-2)
Supplement: Supplementary file 1 — Supplementary Material 1. [file 13018_2025_6176_MOESM1_ESM.docx]

**Appendix 1.** Survey questionnaire asked to surgeons, physiotherapists and occupational therapists

**The management of little finger in Dupuytren's disease**

**1.In which country do you work? ............................................................................................................**

**2.Are you a:**

Surgeon
Physical Therapist
Occupational Therapist

**3. How many patients with Dupuytren's contracture do you treat in one year?**

- 0 to 5
- 5 to 15
- 15 to 30
- 30 to 50
- More than 50

**4. How many patients with little finger Dupuytren's contracture do you treat in one year?**

- 0 to 5
- 5 to 15
- 10 to 30
- 30 to 50
- More than 50

**5. How many are isolated little finger contractures?**

- 0 to 5
- 5 to 15
- 15 to 30
- More than 30

**6. The 5th finger does not necessarily benefit from good results. Rehabilitation, just like surgery, can be delicate and difficult. Do you agree on this point of view?**

Yes No

**7. If you agree with this statement could you briefly describe what difficulties you are experiencing in your own field (surgical or rehabilitation)?**

**...........................................................................................................**

**8. If you don't agree could you explain your answer? ( example. I don't think there are substantial differences with the management of the other fingers)**

**............................................................................................................ 9.Would you be interested to know the results of this survey?**

Yes No

**10.If the answer is yes please feel free to write down your e-mail and I will be glad to share the results with you.**

**............................................................................................................**

**Appendix 2 Key words coding table.**

**2a Key words disagreement answers**

| GOOD RESULTS | good functional results |
| --- | --- |
| SATISFACTION | good patient satisfaction |
| PIPJ | PIP joint involvement |
| SURGERY | depend on surgery skills, surgical techniques |
| DEGREE | depend on degree, depend on stage of deformity |
| EARLY INTERVENTION | depend on early intervention |
| PATIENT COMPLIANCE | depend on patient compliance |

**2b Key words for agreement answers**

| LATE CONSULTING | late consulting, late consultation | |
| --- | --- | --- |
| STRENGHT | force, gripping | |
| ROM DEFICIT | poor range of movement, mobility deficit, stiffness | |
| EXTENSION DEFICIT | | extension deficit, getting final extension, regaining full active extension, inability to achieve full functional extension |
| FLEXION DEFICIT | | locking on the object, coiling deficit, grasp deficit, end of fist, poor flexion, difficulty in recovery flexion, loss of flexion |
| NEUROVASCULAR TRAUMA | | neurovascular deficit, vascular damage, circulatory disturbances, nerve trauma, nerve, and vessels involvement |
| FINGER EXCLUSION | | exclusion of the fingers; integration of the 5th finger in the motor scheme, the finger is less used |
| PAIN | | pain, more frequent pain, pain during motion |
| REHABILITATION | | difficult rehabilitation, rehabilitation not effective, not precise, not prescribed, lack of hand therapists |
| SMALLSIZE | | smaller, small size of structures |
| DEGREE | | stage, deformity, flexion degree |
| MORE FREQUENT | | frequency |
| POOR EDUCATION | | poor patient education |
| WOMEN | | more difficult to treat in women |
| SECONDARY CONTRACTURE | | boutonniere, jaw, MP hyperextension, hook, imbalance of extension and flexion forces, hyper extension, and abduction with shortening of the collateral ligaments, extensor tendons attenuation |
| ANATOMY | | Different anatomy of little finger, anatomical specificity, multiples cords, neurovascular bundles involved in the aponeurotic cords, retrovascular cord, abductor digiti minimi cord, flexor digitorum superficialis absence, stretching of vessels, natural flexion cascade, different neuromuscular control |
| SENSORY DISORDERS | | hypersensivity, hyposensivity, paresthesia, sensitivity to cold |
| ROTATION | | finger rotation |
| SWELLING | | swelling, edema |
| INFLAMMATION | | inflammation, inflammatory reaction, flare reaction |
| SURGERY | | difficult surgery, surgical release |
| RECURRENCE | | |
